# Frequency comb ptychoscopy

**DOI:** 10.1038/s41467-021-24471-4

**Published:** 2021-07-09

**Authors:** David J. Benirschke, Ningren Han, David Burghoff

**Affiliations:** 1 Department of Electrical Engineering, University of Notre Dame, Notre Dame, IN USA; 2 Google LLC, Mountain View, CA USA

**Keywords:** Optical spectroscopy, Frequency combs

## Abstract

Multiheterodyne techniques using frequency combs—radiation sources whose lines are perfectly evenly-spaced—have revolutionized science. By beating sources with the many lines of a comb, their spectra are recovered. Even so, these approaches are fundamentally limited to probing coherent sources, such as lasers. They are unable to measure most spectra that occur in nature. Here we present frequency comb ptychoscopy, a technique that allows for the spectrum of any complex broadband source to be retrieved using a comb. In this approach, the spectrum is reconstructed by unfolding the simultaneous beating of a source with each comb line. We demonstrate this both theoretically and experimentally, at microwave frequencies. This approach can reconstruct the spectrum of nearly any complex source to high resolution, and the speed, resolution, and generality of this technique will allow chip-scale frequency combs to have an impact in a wide swath of new applications, such as remote sensing and passive spectral imaging.

## Abstract (web summary)

Frequency-comb-based multiheterodyne spectroscopy requires that total bandwidth of the measured spectrum covers less than half the comb spacing, which is usually not the case for incoherent spectra. Here, the authors propose a technique that lifts this requirement, and demonstrate it in the microwave regime.

## Introduction

Comb-based multiheterodyne techniques have recently seen a surge in popularity^1^. These techniques take advantage of the heterodyne concept, wherein a known frequency source is mixed with a signal to produce a copy of the signal at lower, intermediate frequencies (IFs). This allows them to be sampled with slower electronics. Frequency combs have broadband coverage and high power per comb tooth, and by beating the many lines of a comb with a signal to be measured, one can measure broadband spectra with high sensitivity in a multiplexed way. For example, dual comb spectroscopy^2–8^ can be used to measure the spectrum of another comb, comb-referenced approaches can measure the spectrum of a laser^9,10^, and vernier spectroscopies^11–13^ can be used to measure the spectra of multi-line lasers. However, all of these techniques require that the resulting spectra do not overlap at IFs, as overlapping IFs create an unavoidable ambiguity in the spectrum. This restricts their use to coherent spectra, such as lasers. This is a major limitation—most spectra found in nature are incoherent. In applications where the signal is broadband and may even be broader than the comb spacing (remote sensing, astronomy, biological systems, etc.), combs can only be used for system calibration. Multiheterodyne techniques are well-suited for measuring the transmission through a system, but not the emission spectrum of a remote source.

This problem is ultimately one of bandwidth. When detecting a complex signal beating with a comb, avoiding ambiguities requires that the total bandwidth of the signal covers less than half the comb spacing. Any broader, and overlap occurs. At the same time, the comb spacing must also be smaller than twice the detector bandwidth (typically a few gigahertz), or else signals may fall too far from comb lines to be detected. Most incoherent optical signals are significantly broader than this limit. Of course, similar bandwidth limitations exist in optical imaging systems, which have limited space-bandwidth products. One particular solution to this problem is to utilize a class of computational methods known as ptychography, which can reconstruct a high-bandwidth image by iteratively stitching together multiple low-bandwidth images^14–18^.

**Fig. 1:**
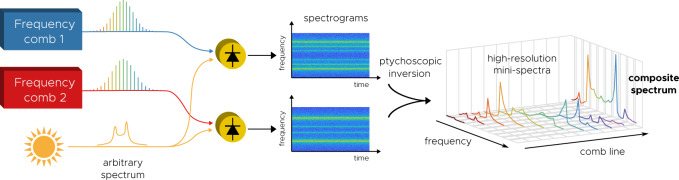
Essential idea of frequency comb ptychoscopy. An arbitrary spectrum is independently beat with two combs, resulting in a pair of spectrograms. By using a ptychoscopic inversion procedure, high-resolution mini-spectra are produced, representing the beating of the source with each comb line. These spectra are limited in bandwidth by photodetection, but their resolution is Fourier-limited. By compositing these spectra, a high-resolution broadband spectrum is produced.

In this work, we introduce *frequency comb ptychoscopy*, a high-resolution multiheterodyne technique that is able to construct the spectrum of arbitrary radiation sources, even broadband sources whose linewidths are much greater than the comb spacing. Inspired by ptychography and by the interferometer-based techniques that disambiguate individual comb lines^19–23^, we show that a signal mixing with a comb can be fully disambiguated using a pair of combs, even when multiple signals overlap at the same IF. The essential idea is shown in Fig. 1. Two spectrograms are produced, and by processing them with an appropriate inversion algorithm, high-resolution mini-spectra are produced. Each mini-spectrum represents the source’s spectrum around a comb line, but its bandwidth is limited to the detector bandwidth. These are then composited to produce a high-resolution, broad spectrum. As in ptychography^14–18^, all of the spectral information is encoded into a narrow bandwidth by the combs. Each version of this measurement has analogs to Fourier spectroscopy and preserves many of its features, such as the throughput and multiplex advantages^24,25^. Though the approach relies on the comb structure, it does not require that the combs have a particular phase profile—the combs can be pulsed^11,13,26,27^ or not^22,26,28–34^. Not only do we derive the inversion algorithm and the methods needed to perform ptychoscopy, but we also present a proof-of-concept measurement at microwave frequencies. Provided suitable detectors and combs are available, this concept can in principle be done at any frequency range.

## Results

### Basic principle

**Fig. 2:**
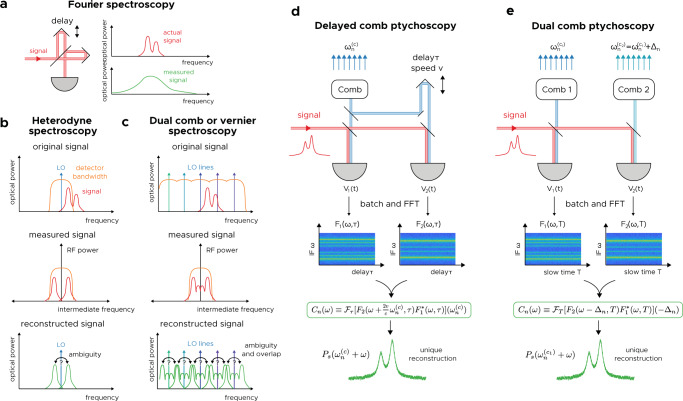
Comparison of various spectroscopic methods with frequency comb ptychoscopy. Various existing spectroscopic approaches (**a**–**c**) and ptychoscopic approaches (**d**, **e**). **a** In Fourier spectroscopy, high-resolution information is lost. **b** In heterodyne spectroscopy, information beyond the detector bandwidth is lost. **c** In vernier spectroscopy, high resolutions can be obtained using a comb, but overlapping signals result in highly ambiguous (non-invertible) spectral reconstructions. **d** In delayed comb ptychoscopy, one comb is split and delayed; both copies are beat with the signal to uniquely reconstruct it. **e** In dual comb ptychoscopy, two combs are independently beat with the signal. Both detectors’ spectrograms are computed and are correlated to reproduce the original signal’s spectrum to high resolution (the inverse of the measurement time divided by the number of comb lines).

Spectroscopic methods generally fall into two categories. *Filter-based* methods use spectral filtering elements like gratings or Michelson interferometers to measure spectra. They can very easily measure broadband spectra, but the challenge with them is the resolution-delay limit—a resolution of Δ*f* requires optical delay of at least *c*/Δ*f*. Achieving resolutions of 1 MHz, for example, would require a system no smaller than 300 m. Therefore, any filter-based spectrometer, such as Fourier spectroscopy, always throws away significant amounts of information at high resolutions (Fig. 2a). By contrast, *heterodyne spectrometers*—essentially radio receivers—intrinsically measure information at high resolutions. By beating a signal with a local oscillator (LO), one can measure spectra with resolutions limited only by the measurement time (Fig. 2b). The challenge with these spectrometers is that their bandwidths are limited to the detector bandwidth. Any information outside this range is lost. Dual comb spectrometers and vernier spectrometers are essentially multiheterodyne techniques, techniques that rely on the beating of a source with the many lines of a comb simultaneously. However, when the signal is broadband and/or complex, information is lost since the spectrum becomes highly ambiguous (Fig. 2c).

Is it possible to combine the attractive features of filter-based methods and of multiheterodyne-based methods? To answer this question, we developed two related methods. The first, referred to as *delayed comb ptychoscopy*, combines a comb with an interferometer to parse out the beating between the unknown signal and a large number of comb lines (Fig. 2d). This is very much in analog to classical Fourier spectroscopy, and was originally an attempt to replicate the success of the SWIFTS technique for waveform reconstruction^22^. The source to be measured is split and is mixed with a comb and a delayed replica of the comb on two separate detectors. As we show in Supplementary Note 1, these signals can be used to uniquely reconstruct the original signal with both heterodyne resolution and broadband coverage.

While delayed comb ptychoscopy is theoretically powerful it is not monolithic, requiring a moving element. Therefore, exploiting the analogy between Fourier spectroscopy and dual-comb spectroscopy, we developed dual-comb ptychoscopy to remedy this issue (Fig. 2e). This is the primary focus of this work. In this case, a second non-identical comb is beat with the signal on a second detector; the combs may or may not be mutually coherent. In each case, the detector signals are digitized and processed into complex spectrograms. (This is done by dividing the data into batches and computing a short-time Fourier transform.) The product of the two spectrograms is computed, and the result is then Fourier transformed again to achieve the final result. Even for a fully incoherent source this correlation function is proportional to the power of the signal (offset from *n*th comb line), which allows it to reconstruct essentially any source. The mathematical details of the two versions differ only slightly, as a Fourier spectrometer can be thought of as Doppler-shifting one comb and generating another^35^.

In the dual comb version, the complex spectrograms *F*_*i*_(*ω*, *T*) are functions of the IF frequency *ω* and the time of each spectrogram *T*. We denote the respective position of the *n*th comb lines as ${\omega }_{n}^{({{{{\rm{c}}}}}_{1})}$ and ${\omega }_{n}^{({{{\rm{{c}}}}_{2}})}\equiv {\omega }_{n}^{({{{{\rm{c}}}}}_{1})}+{{{\Delta }}}_{n}$, where Δ_*n*_ is the separation between corresponding lines. We denote the respective complex amplitudes as ${E}_{n}^{({{{{\rm{c}}}}}_{1})}$ and ${E}_{n}^{({{{\rm{{c}}}}_{2}})}$. We then correlate the two spectrograms and compute *C*_*n*_(*ω*), the Fourier transform of the spectrogram correlation along *T*,

1 $${C}_{n}(\omega )\equiv {{{{\mathcal{F}}}}}_{{{{\rm{T}}}}}[{F}_{{{{\rm{2}}}}}(\omega -{{{\Delta }}}_{n},T){F}_{{{{\rm{1}}}}}^{* }(\omega ,T)](-{{{\Delta }}}_{n}).$$

As we show in Supplementary Note 1, this function is statistically-related to the spectrum of the source *P*_s_ by

2 $$\left\langle {C}_{n}(\omega )\right\rangle ={E}_{n}^{({{{{\rm{c}}}}}_{2})* }{E}_{n}^{({{{{\rm{c}}}}}_{1})}{P}_{{{{\rm{s}}}}}({\omega }_{n}^{({{{{\rm{c}}}}}_{1})}+\omega ).$$

In other words, the spectrum near every comb line can be determined simply by dividing out the amplitude of the dual comb beat signal ${E}_{n}^{({{{{\rm{c}}}}}_{2})* }{E}_{n}^{({{{{\rm{c}}}}}_{1})}$ and stitching the results together. Even though *ω* is small when compared with the total bandwidth of the signal, the spectrum can be measured anywhere comb lines are present. This result holds for both positive and negative IF frequencies as well as overlapping IF frequencies, allowing for complete disambiguation of the signal. This process of constructing a full spectrum from piecewise reconstruction of smaller, folded spectra is reminiscent of ptychographic techniques used in imaging^14–17^. It is for this reason that we refer to this technique as ptychoscopy.

Both the delayed and dual comb versions of ptychoscopy are general, reconstructing the source in practically all cases. The sole situation in which they will not correctly reproduce the spectrum of the signal is when there exist frequencies for which $\left\langle {E}_{{{{\rm{s}}}}}(\omega ){E}_{{{{\rm{s}}}}}^{* }(\omega +n{\omega }_{r})\right\rangle \,\ne\, 0$ over the duration of the measurement (where *ω*_*r*_ is the repetition rate of a comb and n is an integer). Over sufficiently long timescales, this will only fail when the source under consideration is deliberately chosen to match the combs’ repetition rates, for example by attempting to measure another comb with the same spacing. The approach is also general for all types of combs, irrespective of the phase of the comb lines.

### Simulation

**Fig. 3:**
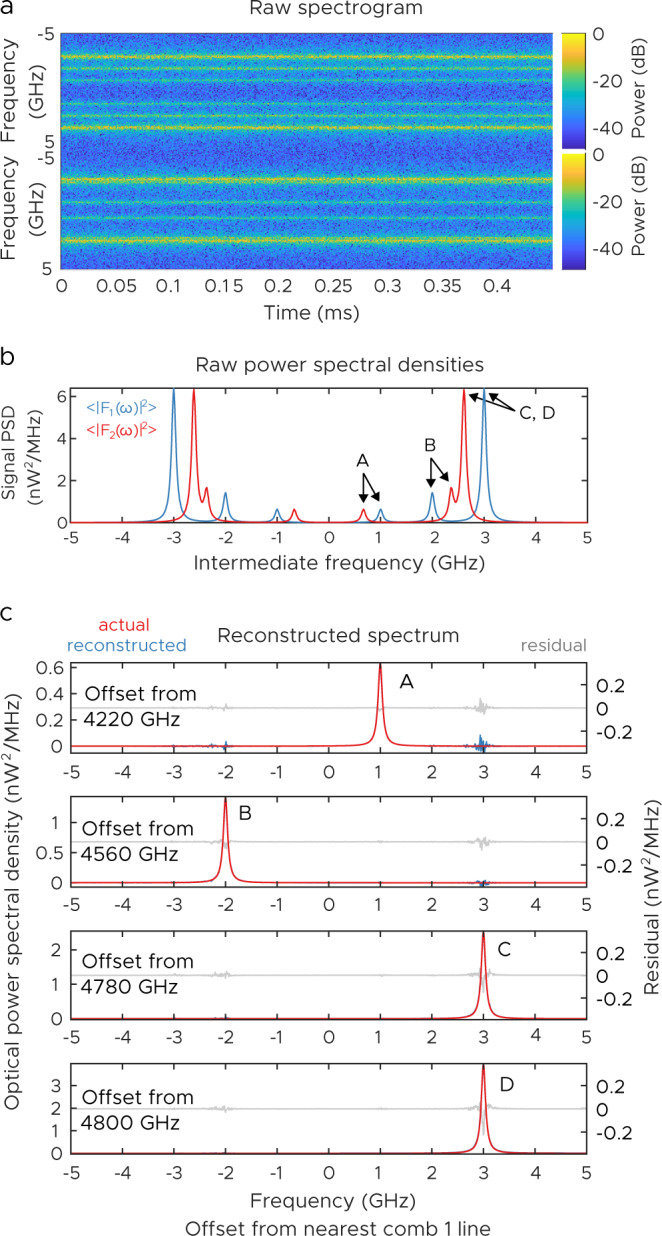
Simulated narrowband terahertz ptychoscopic experiment. **a** Magnitudes of the recorded spectrograms as a function of slow time and IF frequency (10 MHz RBW, 0.45 ms measurement time). **b** Raw signal power spectral densities, with contributions from beating with various lines labeled. **c** Reconstructed signals calculated from Eq. (2), along with the actual spectrum.

As a relevant example, we first consider a simulation of a complex terahertz spectrum consisting of several lines, similar to the type of signal that is highly relevant for astronomy (for example, in measuring the spectral line energy distribution of carbon monoxide^36^). For astronomical signals, which have a mix of both narrowband and broadband features, being able to simultaneously measure high-resolution spectra with broadband coverage would be valuable—there is a significant need for multi-line detection in many astronomical missions, and observation time on suborbital and space missions is extremely expensive. We consider signals in the range of 4 to 5 THz and consider the dual comb version of the measurement. Our combs are assumed to span 4–5 THz with repetition rates of 10 GHz (typical parameters for quantum cascade laser combs^21,37^). Our signals are fairly broadband—with 100 MHz full-width half maximums (FWHMs)—and are generated numerically using a phase random walk process. A list of the line strengths and locations is shown in Table 1. Since our method is radiometric in nature, probing sources rather than absorption line strengths, the line strengths represent emitted power. These lines are chosen to illustrate the power of the technique. Lines A, C, and D appear at positive IFs (relative to the nearest comb line), while line B appears at a negative IF. With a single LO line and one detector, it is impossible to distinguish positive IFs from negative IFs. Furthermore, lines C and D appear at the exact same IF, which means that distinguishing them is impossible with all prior vernier-like techniques. In this case, the lines are relatively broadband but are much narrower than the comb spacing. The corresponding magnitude of the two spectrograms is shown in Fig. 3a.

**Table 1:** Simulated spectral parameters corresponding to Fig. 3.

Line	Frequency (GHz)	Emission power (pW)	Offset from comb 1 (GHz)	Offset from comb 2 (GHz)
A	4211	0.1	1	0.669
B	4558	0.225	−2	−2.366
C	4783	0.4	3	2.612
D	4803	0.625	3	2.610

Lines of the spectrum considered. Comb 1 spans 4-5 THz with a repetition rate of 10 GHz. Comb 2 has a repetition rate of 10 GHz+1 MHz and has an additional offset of 0.3 GHz. Comb lines have a power of 1 mW per tooth.

Individually, the two spectrograms appear as a noisy version of the average power spectral density of the signal on the two detectors. This “noise” actually arises from the incoherent nature of our signals, and it is the hidden correlations of these two signals that give rise to our computed result. Similarly, the average power spectral densities of the two signals are shown in Fig. 3b. They contain peaks from all lines beating with the combs—the result is a complex zoo of overlapping spectra.

Figure 3c shows the results of our correlation calculation. For each comb line, we compute the real part of *C*_*n*_(*ω*) and plot the result for comb lines that are near signal lines. We also show a theoretical prediction of $\left|{E}_{n}^{({{{{\rm{c}}}}}_{2})* }{E}_{n}^{({{{{\rm{c}}}}}_{1})}\right|{P}_{{{{\rm{s}}}}}({\omega }_{n}^{({{{{\rm{c}}}}}_{1})}+\omega )$. The agreement between the two is excellent. For example, looking at the spectrum near line A (the weakest line), very little evidence of other lines is present despite the fact that much larger signals are beating at ±2 GHz and ±3 GHz on the raw spectrograms. Only small zero-mean image noise remains in the result. Note also that line B correctly appeared at −2 GHz, not 2 GHz. Though *F*_1_ and *F*_2_ must be conjugate-symmetric, *C*_*n*_ is not. Finally, note that lines C and D are also correctly distinguished despite the fact that they overlap entirely with respect to both combs 1 and 2.

**Fig. 4:**
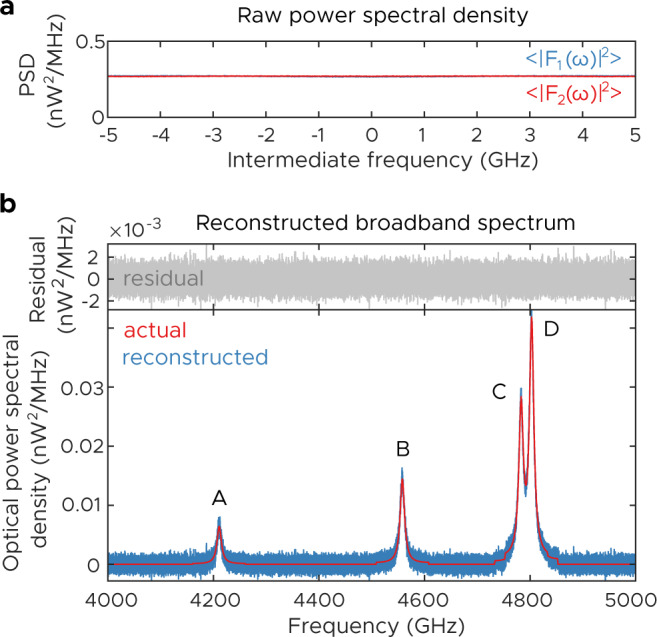
Simulated broadband terahertz ptychoscopic experiment. **a** Raw power spectral densities of the recorded signals when the spectrum has components broader than the comb spacing. They are essentially flat, having no discernible peaks. **b** Spectral reconstruction and true spectrum (10 MHz RBW, 9 ms measurement time).

These results are valid for any source, even incoherent sources. To illustrate this, we increase the FWHM of the lines shown above to 10 GHz—the comb repetition rate—and plot the reconstructions. With linewidths this broad, the entire IF span is filled with a relatively flat spectrum (see Fig. 4a). Since the true spectrum does not have narrow features, we plot the full reconstructed spectrum in Fig. 4b. Once again, the results are in good agreement with the theoretical prediction. Note that because lines C and D are only 2*f*_*r*_ apart, their lineshapes overlap both in real frequency and in intermediate frequency. Although in this case there is very little information to be gained from going to resolution bandwidths this narrow, in the general case where the spectrum’s features are totally unknown the ability to perform high-resolution measurements is critical.

### Experimental results

To prove our concept, we present ptychoscopic reconstruction of microwave signals, using the dual comb version outlined in Fig. 2. Note that nothing in our approach relies on the spectral range of interest or on the type of source or detector. (Similarly, the first dual-comb spectroscopy was conducted using electronic sources in the range of 50–300 GHz^38^; extending it to the optical domain required no change in principle, only changes in the combs and detectors.) With this in mind, there are two key metrics needed to judge the proposed method as compared to previous dual-comb spectroscopic techniques. First, is it possible to simultaneously achieve heterodyne resolution and broadband coverage when the comb covers hundred of spectral lines? Second, can an incoherent spectrum much broader than a single repetition rate be faithfully reconstructed? To date; no other dual comb technique has been able to achieve both goals simultaneously; we will demonstrate each capability presently.

In order to correct for systemic experimental errors, several calibrations and corrections must be conducted. First, an IF calibration is done to account for the detector’s response. A broad flat spectrum spanning multiple comb lines is used to generate a flat IF spectrum, and any deviation from flatness is attributed to the detector response and is corrected. Second, the signals are phase-corrected using the method described in Supplementary Note 3. Last, the signal was normalized to correct for comb line power variations.

**Fig. 5:**
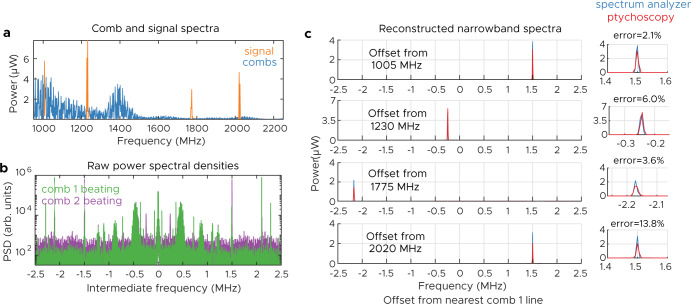
Narrowband microwave ptychoscopic experiment. Ptychoscopic reconstruction of narrowband signals that span an octave. **a** Spectrum of the signal that is to be measured, along with the spectrum of combs’ lines. **b** Raw power spectral density recorded on the two detectors, showing a complex mix of spectra. **c** Ptychoscopic reconstruction of data and of real signals, along with the error of the integrated power. Note that the two methods have different instrument lineshapes and resolution bandwidths—this results in different peak heights, but the integrated powers are similar (see errors).

For the first measurement, we use combs of repetition rates 5 MHz and 4.997 MHz, respectively, and detect them with a bandwidth of 2.5 MHz. We generate our spectrum by combining 4 different single-frequency synthesizers, and the result is a simulacrum of the previous terahertz simulations. The lines are located at, 1006.5, 1229.7, 1772.8, and 2021.5 MHz, and our spectrum spans 203 comb lines and is octave-spanning. In addition, the first and fourth comb line were chosen to both be offset from their nearest comb line by 1.5 MHz. Their successful resolution illustrates the ability of our technique to differentiate signals that overlap in IF. The results of this measurement are reported in Fig. 5. The signal was generated using a 40 ms acquisition time with a resolution of 6.8 kHz. Both the reconstructed signal and the ground truth measured via an RF spectrum analyzer are shown. While there are some discrepancies in the exact height and shape of the peaks due to differences in the resolution of the two measurements, the power of each line is correctly recovered, giving respective errors of 2.1, 6.0, 3.6, and 13.8%. (The higher error of the last measurement is due to the weakness of the comb sources at 2 GHz.)

**Fig. 6:**
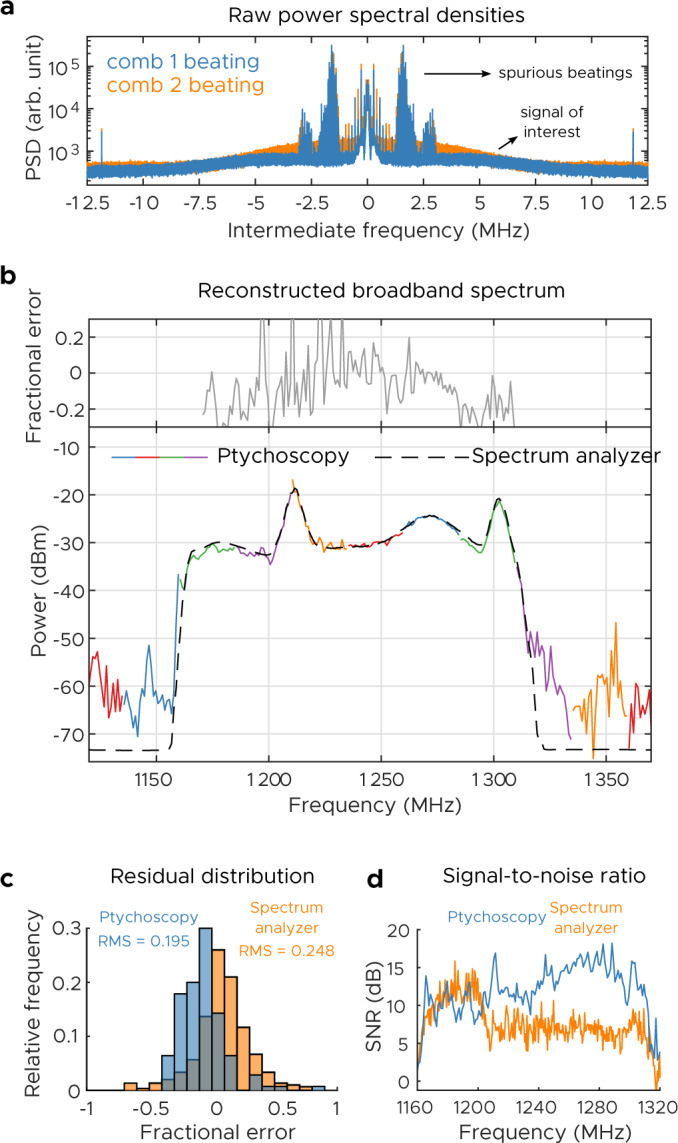
Broaband microwave ptychoscopic experiment. **a** Raw power spectral densities of the recorded signals. **b** Spectral reconstruction by ptychoscopy and true spectrum. The multi-color curve is the reconstruction where each color corresponds to a different comb line. The dashed line represents the ground truth, as measured on an RF spectrum analyzer (averaged). **c** Comparison of the distribution of residuals between the ptychoscopic measurement (over the valid range) and the spectrum analyzer measurement when the measurements are taken with the same RBW and measurement time. **d** Comparison of the two signal-to-noise ratios (SNRs). The ptychoscopic measurement is better on account of the non-ergodicity of the incoherent waveform.

While the previous reconstruction was correct, because it consisted of a few discrete lines it could have been inverted using vernier techniques^13^. It does not display the full power of ptychoscopy. To do this, we consider a highly degenerate spectrum where the intermediate frequencies overlap multiple times. For this measurement, we utilize combs with repetition rates of 25 MHz and 24.95 MHz, respectively. We choose a frequency-modulated (FM) voltage controlled oscillator (VCO) as the input signal. In order to generate an interesting incoherent spectrum, we FM modulate the VCO using a random noise voltage with a desired distribution (see Supplementary Note 5 for more details). This random modulation is chosen to generate an incoherent spectrum that spans multiple comb lines, approximately eight repetition rates wide, and consists of both broad and narrow features (Fig. 6b). First, the spectrum is measured with a spectrum analyzer (100 averages, 10 ms acquisition time, 1 MHz resolution bandwidth, resulting in 25 points per repetition rate). Ptychoscopy is performed on this signal, and Fig. 6a shows the raw power spectral densities measured on each dual comb channel. The spikes below 3 MHz represent residual beating between the two combs due to crosstalk; the signal of interest actually appears as a weak baseline spread throughout the whole IF range. The raw spectra are essentially flat and do not resemble the true spectrum in any way; they also have poor signal-to-noise ratio (under 5 dB). This is due to the fact that the underlying signals are much broader than the comb repetition rate. Nevertheless, using this data and following the reconstruction procedure yields a spectrum that agrees well with the ground truth. The spectrum has also been accurately reconstructed over eight repetition rates, despite the fact that we are only using information up to half the combs’ repetition rates (12.5 MHz).

It is interesting to compare the signal-to-noise ratio (SNR) of our method with that of a conventional RF spectrum analyzer (Fig. 6c, d). One may expect that because the spectrum analyzer utilizes a tunable LO, its SNR would be superior. However, one finds that for the same resolution bandwidth (1 MHz) and measurement time (10 ms), the ptychoscopic reconstruction is actually superior—having less root-mean-square (RMS) error, thinner tails, and much higher SNR. This is ultimately because the VCO is not a true stationary incoherent source. It switches frequency randomly every 4 μs, and the result of this is that single sweeps of the spectrum analyzer produce highly variable results. Ptychoscopy, by contrast, lacks this problem entirely. It is capable of accurately resolving spectra even in single-shot mode, as all of the signal is always recorded.

## Discussion

As this technique extends multiheterodyne spectroscopy to the case of incoherent spectra, it maintains many of the appealing features of other multiheterodyne techniques. The dual comb version is similar to other dual comb techniques in that the ultimate time resolution of this measurement is determined by the ability to resolve different dual comb beat signals^11,12^. As such, its time resolution is 1/Δ*f*_r_ and can easily be within the range of microseconds (contingent on the signal-to-noise ratio). While the delayed comb version is limited by the mechanical speed of the delay element, chip-scale combs such as quantum cascade lasers and microresonator combs typically have repetition rates in the ten of GHz range, meaning that resolving their features requires only a travel distance of a few millimeters. This is conceivably within the range of 100 ms.

Next, we consider frequency resolution. For perfect comb sources the resolution bandwidth (RBW) is determined by the batch length according to RBW$=\frac{1}{{N}_{{{{\rm{t}}}}}{{\Delta }}t}$, where Δ*t* is the sample interval and *N*_t_ is the number of samples per batch. However, resolving the dual comb beat term requires that there be at least as many batches (*N*_T_) as the number of comb lines (*N*_c_). If *T* is the total measurement time *T* ≡ *N*_T_*N*_t_Δ*t*, then this requires that ${{{\rm{RBW}}}}\, > \,\frac{{N}_{{{{\rm{c}}}}}}{T}$. In other words, the resolution is limited by the number of comb lines divided by the measurement time. This is worse than what is achievable with isolated lines (which allows for resolutions of 1/*T*^11^) because knowing that the line is isolated requires prior knowledge of the signal. Effectively, we traded some resolution for the ability to distinguish lines that overlap in the IF. Practically speaking this is only relevant for phase-stable combs; for free-running combs it is typically the linewidths of the combs themselves that set the technique’s resolution.

In terms of sensitivity, this approach has many similarities to traditional Fourier spectroscopy. Because the entire signal is measured at once and is not demultiplexed, the multiplex advantage^24^ is maintained. Additionally, because the system does not require a single-mode source, the throughput advantage^25^ is maintained. A detailed discussion is given in Supplementary Note 2; for additive noise, the total noise is the sum of image noises from every signal sharing an IF frequency. When compared with a tunable LO of the same total power (i.e., a conventional radiometer), in best case scenario of non-overlapping signals the sensitivities are identical. In the worst case scenario of broadband spectra the RMS sensitivity is $\sqrt{{N}_{{{{\rm{c}}}}}}$ worse, as the LO power of the individual lines is effectively divided by *N*_c_. While this is problematic for certain astronomical applications where quantum-limited sensitivity is required, for dynamically-varying sources the dual comb version of this approach will be considerably faster than a widely-tunable LO (which typically requires moving parts) or a Fourier spectrometer. Detector nonlinearity will further degrade the sensitivity, see Supplementary Note 4.

Like all comb-based spectroscopies, this approach suffers in the presence of comb phase noise. While the delayed comb version is fairly immune to such effects, the dual comb version is highly susceptible. Still, like dual comb spectroscopy^39–44^, it can be corrected after the fact. This procedure is detailed in Supplementary Note 3, and essentially requires that a standard dual comb measurement (measuring the beating of the two combs) is performed. This additional measurement can also be used to calibrate the amplitude, as a dual comb measurement will produce the beat signal ${E}_{n}^{({{{{\rm{c}}}}}_{2})}{E}_{n}^{({{{{\rm{c}}}}}_{1})* }$ that is needed to normalize the result.

Next, we place this result in the context of earlier work, in particular with respect to vernier spectroscopy^11–13^. While the delayed comb version of this approach has not been demonstrated in any capacity (to our knowledge), the dual comb version is experimentally very similar to vernier spectroscopy. Previous work did not attempt to perform spectroscopy of incoherent sources, focusing on swept-source diode lasers^11–13^ and fiber mode-locked lasers^13^. The key distinction is that in all of these cases, individual lines can be isolated provided they do not occur at the same IF frequency, and correlation maximization procedures can be used to find the comb order number of each line. However, these approaches require signals that fill a vanishingly small fraction of the repetition rate (even if they do so discontinuously), as continuous overlapping spectra from different orders are not compatible with this approach. By contrast, our work shows that eightfold degeneracy can be resolved (Fig. 6). Though ref. ^11^ did describe a method of reconstructing continuous spectra like ours, this came at the cost of resolution—it could only resolve one point per *f*_r_. By contrast, our results in Figs. 5 and 6 resolve up to 750 and 25 points per *f*_r_, respectively. Having said that, Eq. (2) is immediately applicable to these previous results, providing another way to analyze them.

Finally, we put our work in context with other broadband methods. While it is clear that our method provides broader reconstruction bandwidth than heterodyne techniques (at the cost of reduced resolution), classic grating spectrometers are of course also capable of broadband incoherent measurements. It is well-known that for a given resolution, a grating spectrometer provides the ideal sensitivity since every photon is captured and utilized for signal generation^45,46^. This is why they out-compete interferometric methods such as FTIR in sensitivity (where a portion of the power is thrown out). In addition, they are not limited by fundamental quantum noise, as heterodyne detection is. The key challenge with these spectrometers is in the resolutions they can obtain for a given size. The resolving power of a spectrometer can be defined to be *f*/Δ*f*, where *f* is the frequency and Δ*f* the resolution. For the measurement of Fig. 5, the worst case resolving power obtained is 1006 MHz / 6.8 kHz = 150,000. For a grating spectrometer, the required size is the product of the resolving power and the desired wavelength. If one desires a grating spectrometer with an equivalent resolving power at 4.5 THz, the linear dimensions of the spectrometer must be no smaller than 10 m. Such a spectrometer would almost certainly not be monolithic, requiring very slow moving parts. While a grating spectrometer could always have better sensitivity in theory, they become highly impractical for dynamically-varying spectra, and particularly at longer wavelengths.

In conclusion, we have demonstrated frequency comb ptychoscopy, a technique that is able to unravel the spectrum of an arbitrary radiation source using combs. This can be done either using two separate combs or one comb with a copy that has been delayed. Even when the beating of the two combs appears random and chaotic, a periodically-varying correlation persists in the complex spectrograms, and this can be exploited to infer the spectrum. This result is compatible with all existing comb technologies and will allow for passive high-speed spectroscopy of essentially any dynamically-varying electromagnetic source. For example, one can imagine applications in reaction kinetics^47^, in biology^48,49^, pharmaceuticals^50^, or millimeter-wave systems^51^.

## Supplementary information

Supplementary Information

## Data Availability

The data that support the findings of this study are available in Figshare with the identifier [10.6084/m9.figshare.c.5489250].

## Code availability

The code that support the findings of this study are available in Figshare with the identifier [10.6084/m9.figshare.c.5489250].

## References

[1] Picqué N, Hänsch TW (2019). Frequency comb spectroscopy. Nat. Photonics.

[2] Keilmann F, Gohle C, Holzwarth R (2004). Time-domain mid-infrared frequency-comb spectrometer. Opt. Lett..

[3] Coddington I, Newbury N, Swann W (2016). Dual-comb spectroscopy. Optica.

[4] Wang Y, Soskind MG, Wang W, Wysocki G (2014). High-resolution multi-heterodyne spectroscopy based on Fabry-Perot quantum cascade lasers. Appl. Phys. Lett..

[5] Villares G, Hugi A, Blaser S, Faist J (2014). Dual-comb spectroscopy based on quantum-cascade-laser frequency combs. Nat. Commun..

[6] Yang Y (2016). Terahertz multiheterodyne spectroscopy using laser frequency combs. Optica.

[7] Yu M (2018). Silicon-chip-based mid-infrared dual-comb spectroscopy. Nat. Commun..

[8] Suh M-G, Yang Q-F, Yang KY, Yi X, Vahala KJ (2016). Microresonator soliton dual-comb spectroscopy. Science.

[9] Del’Haye P, Arcizet O, Gorodetsky ML, Holzwarth R, Kippenberg TJ (2009). Frequency comb assisted diode laser spectroscopy for measurement of microcavity dispersion. Nat. Photonics.

[10] Bartalini S (2014). Frequency-comb-assisted terahertz quantum cascade laser spectroscopy. Phys. Rev. X.

[11] Giorgetta FR, Coddington I, Baumann E, Swann WC, Newbury NR (2010). Fast high-resolution spectroscopy of dynamic continuous-wave laser sources. Nat. Photonics.

[12] Coddington I, Giorgetta FR, Baumann E, Swann WC, Newbury NR (2012). Characterizing Fast Arbitrary CW Waveforms With 1500 THz/s Instantaneous Chirps. IEEE J. Sel Topics Quant. Electr..

[13] Yang Q-F (2019). Vernier spectrometer using counterpropagating soliton microcombs. Science.

[14] Rodenburg, J. M. Ptychography and Related Diffractive Imaging Methods. In Hawkes, editor, *Advances in Imaging and Electron Physics*, volume 150, pages 87–184. (Elsevier, January 2008).

[15] Pfeiffer F (2018). X-ray ptychography. Nat. Photonics.

[16] Zheng G, Horstmeyer R, Yang C (2013). Wide-field, high-resolution Fourier ptychographic microscopy. Nat. Photonics.

[17] Tian L (2015). Computational illumination for high-speed in vitro Fourier ptychographic microscopy. Optica.

[18] Sun J, Zuo C, Zhang L, Chen Q (2017). Resolution-enhanced Fourier ptychographic microscopy based on high-numerical-aperture illuminations. Sci. Rep..

[19] Mandon J, Guelachvili G, Picqué N (2009). Fourier transform spectroscopy with a laser frequency comb. Nat. Photonics.

[20] Hugi A, Villares G, Blaser S, Liu HC, Faist J (2012). Mid-infrared frequency comb based on a quantum cascade laser. Nature.

[21] Burghoff D (2014). Terahertz laser frequency combs. Nat. Photonics.

[22] Burghoff D (2015). Evaluating the coherence and time-domain profile of quantum cascade laser frequency combs. Optics Express.

[23] Han Z, Ren D, Burghoff D (2020). Sensitivity of SWIFT spectroscopy. Optics Expr..

[24] Fellgett, P. B. *The Theory of Infrared Sensitivities and Its Application to Investigations of Stellar Radiation in the Near Infra-Red*. (1951).

[25] Jacquinot P (1958). Caractères communs aux nouvelles méthodes de spectroscopie interférentielle ; Facteur de mérite. Journal de Physique et le Radium.

[26] Hillbrand J (2020). In-phase and anti-phase synchronization in a laser frequency comb. Phys. Rev. Lett..

[27] Bagheri M (2018). Passively mode-locked interband cascade optical frequency combs. Sci. Rep..

[28] Singleton M, Jouy P, Beck M, Faist J (2018). Evidence of linear chirp in mid-infrared quantum cascade lasers. Optica.

[29] Dong M, Cundiff ST, Winful HG (2018). Physics of frequency-modulated comb generation in quantum-well diode lasers. Phys. Rev. A.

[30] Henry N, Burghoff D, Yang Y, Hu Q, Khurgin JB (2017). Pseudorandom dynamics of frequency combs in free-running quantum cascade lasers. Opt. Eng..

[31] Cappelli F (2019). Retrieval of phase relation and emission profile of quantum cascade laser frequency combs. Nat. Photonics.

[32] Del’Haye P (2015). Phase steps and resonator detuning measurements in microresonator frequency combs. Nat. Commun..

[33] Metcalf AJ, Torres-Company V, Leaird DE, Weiner AM (2013). High-power broadly tunable electrooptic frequency comb generator. IEEE J. Selected Topics Quant. Electr..

[34] Burghoff D (2020). Unraveling the origin of frequency modulated combs using active cavity mean-field theory. Optica.

[35] Mandon J, Guelachvili G, Picqué N (2009). Fourier transform spectroscopy with a laser frequency comb. Nat. Photonics.

[36] Mashian N (2015). High-J CO SLEDs in nearby infrared bright galaxies observed by Herschel/PACS. The Astrophys. J..

[37] Faist J (2016). Quantum cascade laser frequency combs. Nanophotonics.

[38] Van der Weide DW, Murakowski J, Keilmann F (2000). Gas-absorption spectroscopy with electronic terahertz techniques. IEEE Trans. Microwave Theory Tech..

[39] Ideguchi T, Poisson A, Guelachvili G, Picqué N, Hänsch TW (2014). Adaptive real-time dual-comb spectroscopy. Nature Communications.

[40] Burghoff D, Yang Y, Hu Q (2016). Computational multiheterodyne spectroscopy. Sci. Adv..

[41] Hébert NB (2017). Self-corrected chip-based dual-comb spectrometer. Opt. Expr..

[42] Sterczewski LA, Westberg J, Wysocki G (2019). Computational coherent averaging for free-running dual-comb spectroscopy. Opt. Expr..

[43] Sterczewski ŁA, Przewłoka A, Kaszub W, Sotor J (2019). Computational Doppler-limited dual-comb spectroscopy with a free-running all-fiber laser. APL Photonics.

[44] Burghoff D, Han N, Shin JH (2019). Generalized method for the computational phase correction of arbitrary dual comb signals. Opt. Lett..

[45] Zmuidzinas, J. et al. Coherent Detection and SIS Mixers. In *Far-IR, Sub-Mm & MM Detector Technology Workshop*, page 68 (2002).

[46] Zmuidzinas, J. The role of coherent detection. *Proceedings of the Second Workshop on New Concepts for Far-Infrared and Submillimeter Space Astronomy* (2004).

[47] Pinkowski NH (2020). Dual-comb spectroscopy for high-temperature reaction kinetics. Meas. Sci. Technol..

[48] Karpov M, Pfeiffer MHP, Liu J, Lukashchuk A, Kippenberg TJ (2018). Photonic chip-based soliton frequency combs covering the biological imaging window. Nat. Commun..

[49] Klocke JL (2018). Single-shot sub-microsecond mid-infrared spectroscopy on protein reactions with quantum cascade laser frequency combs. Anal. Chem..

[50] Sterczewski LA (2019). Terahertz hyperspectral imaging with dual chip-scale combs. Optica.

[51] Hoffmann C, Russer P (2011). A real-time low-noise ultrabroadband time-domain EMI measurement system up to 18 GHz. IEEE Trans. Electromagn. Compatib..

