# Author Correction: Frequency comb ptychoscopy

**DOI:** 10.1038/s41467-023-38450-4

**Published:** 2023-05-16

**Authors:** David J. Benirschke, Ningren Han, David Burghoff

**Affiliations:** 1 Department of Electrical Engineering, University of Notre Dame, Notre Dame, IN USA; 2 Google LLC, Mountain View, CA USA

**Keywords:** Optical spectroscopy, Frequency combs

Correction to: *Nature Communications* 10.1038/s41467-021-24471-4, published online 9 July 2021

In this article the funding from the Office of Naval Research (ONR) was omitted.

The original article has been corrected.

