# Supplementary material for: Frequency comb ptychoscopy
Source: Nat Commun. 2021 Jul 9;12:4244. doi: 10.1038/s41467-021-24471-4 (PMC8270995; doi:10.1038/s41467-021-24471-4)
Supplement: Supplementary file 1 — Supplementary Information [file 41467_2021_24471_MOESM1_ESM.pdf]

Supplementary information for "Frequency comb ptychoscopy"

David Benirschke[1], Ningren Han[2], and David Burghoff[1]
[1] Department of Electrical Engineering,
University of Notre Dame, Notre Dame, IN 46656
[2]Google LLC, Mountain View, California 94043

SUPPLEMENTARY NOTE 1: DETAILED DERIVATION

To show that equation (2) of the main text holds, we will derive the result for the dual comb version first and extend it to the delayed comb version. All electric fields are expressed as a superposition of exponentials as

$$E_{c_i}(t) = \sum_n E_n^{(c_i)} e^{i\omega_n^{(c_i)} t} \quad (1)$$

$$E_s(t) = \sum_m E_m^{(s)} e^{i\omega_m^{(s)} t} \quad (2)$$

where $E_{c_i}(t)$ is the field of the ith comb and $E_s(t)$ is the field of the signal to be measured. For convenience the signal is represented as a summation rather than an integral. In a heterodyne measurement, the raw signal that is recorded is

$$\begin{aligned} S_i(t) &= \frac{1}{2}\left|E_{c_i}(t) + E_s(t)\right|^2 \sim E_{c_i}^* E_s \\ &= \sum_{n,m} E_n^{(c_i)*} E_m^{(s)} e^{i(\omega_m^{(s)} - \omega_n^{(c_i)})t} \end{aligned} \quad (3)$$

where we neglect the intracomb beat terms (which occur only at vanishingly-narrow multiples of the repetition rate) and the intrasignal beat terms (which are assumed to be small).

We assume that our detector has a bandwidth larger than $f_r/2$ (half the comb repetition rates) and that the signal is digitized with a sample period Δt sufficiently small to avoid aliasing. In the spirit of spectrograms, time is divided into two separate time axes—the fast time t_i and the slow time T_j—such that the total time is given by $t = t_i + T_j$. The data is similarly divided into batches that are $N_t \Delta t$ long (which determines the resolution bandwidth). In other words, T_j can be taken as $T_j = (j-1)N_t\Delta t$, where j is an integer.

Dual comb version. First, we calculate the signals in terms of the FFT IF frequencies $\omega_k \equiv \frac{2\pi}{N_t \Delta t}k$. For the FFT, we use the convention that $\mathcal{F}[f](\omega_k) \equiv \frac{1}{N_t}\sum_i e^{-i\omega_k t_i} f_i$. For comb 1, we find that the spectrogram $F_1(\omega_k, T_j)$ is given by

$$\begin{aligned} F_1(\omega_k, T_j) &= \frac{1}{N_t}\sum_i e^{-i\omega_k t_i} S_1(t_i + T_j) \\ &= \frac{1}{N_t}\sum_{i,n,m} E_n^{(c_1)*} E_m^{(s)} e^{i(\omega_m^{(s)} - \omega_n^{(c_1)} - \omega_k)t_i} \\ &\qquad \times e^{i(\omega_m^{(s)} - \omega_n^{(c_1)})T_j}. \end{aligned} \quad (4)$$

Because our resolution bandwidth is determined by our batch length (RBW= $\frac{1}{N_t\Delta t}$), in order to proceed we make an approximation in which our signal frequencies are all an integer number of RBWs away from comb 1, i.e. $\omega_m^{(s)} - \omega_n^{(c_1)} = \frac{2\pi}{N_t\Delta t} l$ for some integer l. As a result, $e^{i(\omega_m^{(s)} - \omega_n^{(c_1)})T_j} = 1$, and

$$\begin{aligned} F_1(\omega_k, T_j) &= \sum_n E_n^{(c_1)*} \frac{1}{N_t} \sum_i e^{-i(\omega_n^{(c_1)} + \omega_k)t_i} \\ &\qquad \times \sum_m E_m^{(s)} e^{i\omega_m^{(s)} t_i} \\ &= \sum_n E_n^{(c_1)*} E_s(\omega_n^{(c_1)} + \omega_k), \end{aligned} \quad (5)$$

where in the last line we used the definition of the FFT twice. Due to our finite resolution bandwidth approximation, the spectrogram would appear to be constant in T. However, once this approximation has been made it cannot be modified when computing the same quantity for comb 2. Performing a $1 \to 2$ substitution, one finds that

$$e^{i(\omega_m^{(s)} - \omega_n^{(c_2)})T_j} = e^{i(\omega_m^{(s)} - \omega_n^{(c_1)} - \Delta_n)T_j} = e^{-i\Delta_n T_j} \quad (6)$$

$$F_2(\omega_k, T_j) = \sum_n E_n^{(c_2)*} E_s(\omega_n^{(c_2)} + \omega_k) e^{-i\Delta_n T_j}. \quad (7)$$

Thus, while F_1 is stationary in time, F_2 is not, and in fact beats periodically at the dual comb frequencies Δ_n. Provided the data has been recorded long enough to resolve individual beat frequencies (i.e., that the data is at least recorded for $1/\Delta f_r = 1/|f_{r_2} - f_{r_1}|$), these beatings can be resolved. For simplicity, we assume that the number of batches N_T has been chosen to ensure that $N_T N_t \Delta t = 1/\Delta f_r$, which will resolve exactly one dual comb beat tooth.

Finally, we compute the Fourier transform of the correlation function and its expectation value:

$$C_n(\omega_k) \equiv \mathcal{F}_T[F_2(\omega_k - \Delta_n, T_j) F_1^*(\omega_k, T_j)](-\Delta_n) \quad (8)$$

$$\begin{aligned} \langle C_n(\omega_k)\rangle &= \frac{1}{N_T}\sum_{j,l,m} E_l^{(c_2)*} E_m^{(c_1)} e^{i(\Delta_n - \Delta_l)T_j} \\ &\quad \times \left\langle E_s(\omega_l^{(c_1)} + \Delta_l - \Delta_n + \omega_k) E_s^*(\omega_m^{(c_1)} + \omega_k)\right\rangle \end{aligned} \quad (9)$$

Because the number of batches was chosen to be an integer number of $1/\Delta f_r$, the summation $\frac{1}{N_T}\sum_j e^{i(\Delta_n - \Delta_l)T_j}$ is a summation over roots of unity and vanishes unless

$n = l$, leaving

$$\langle C_n(\omega_k)\rangle = \sum_m E_n^{(c_2)*} E_m^{(c_1)} \times \left\langle E_s(\omega_n^{(c_1)} + \omega_k) E_s^*(\omega_m^{(c_1)} + \omega_k) \right\rangle \quad (10)$$

This result is general for any source. For the vast majority of sources we can make an additional assumption, which is that the long-term correlation between components of frequencies spaced by the repetition rate of the comb vanishes. This assumption is essentially valid for any source that is not a comb of the same spacing as either of the LO combs. With this additional assumption we can eliminate the cross terms, leaving

$$\langle C_n(\omega_k)\rangle = E_n^{(c_2)*} E_n^{(c_1)} \left\langle \left| E_s(\omega_n^{(c_1)} + \omega_k) \right|^2 \right\rangle. \quad (11)$$

By calculating every C_n, the signal's power relative to a comb line can always be extracted.

Delayed comb version. For the delayed comb, the analysis is similar. The analysis for comb 1 is fully identical (letting $E_n^{(c_1)} \to E_n^{(c)}$), and the analysis for comb 2 is found by setting $E_n^{(c_2)} \to E_n^{(c)} e^{-i\omega_n^{(c)}\tau}$ and $\Delta_n = 0$. However, a subtlety arises when delay is a linear function of lab time (i.e., a linear scan is performed rather than a step scan). Because delay changes during the batch, this has the same effect as Doppler shifting the IF frequencies in a manner similar to a nonzero Δ_n. One should therefore proceed as before, but using the explicit mapping $\tau \to \frac{2v}{c}(t_i + T_j)$. This results in

$$F_2(\omega_k, T_j) = \sum_n E_n^{(c)*} E_s\left(\omega_n^{(c)} - \omega_n^{(c)}\frac{2v}{c} + \omega_k\right) e^{i\omega_n^{(c)}\frac{2v}{c}T_j} \quad (12)$$

By comparing this to the dual comb version of the same result, we find that $-\omega_n^{(c)}\frac{2v}{c}$ has replaced Δ_n. If we now define $\tau_j \equiv \frac{2v}{c}T_j$ and make the appropriate substitutions into our definition of C_n, we find that we must instead calculate

$$C_n(\omega_k) \equiv \mathcal{F}_\tau[F_2(\omega_k + \omega_n^{(c)}\frac{2v}{c}, \tau_j)F_1^*(\omega_k, \tau_j)](\omega_n^{(c)}) \quad (13)$$

which results in

$$\langle C_n(\omega_k)\rangle = \left|E_n^{(c)}\right|^2 \left\langle \left| E_s(\omega_n^{(c)} + \omega_k) \right|^2 \right\rangle. \quad (14)$$

Example: single-line reconstruction

When only one line is present in the signal, vernier spectroscopy is sufficient for reconstruction. However, it is still instructive to see how ptychoscopy reconstructs such signals. Consider the dual comb case and consider a signal consisting of a single line, $E_s e^{i\omega_s t}$, which beats

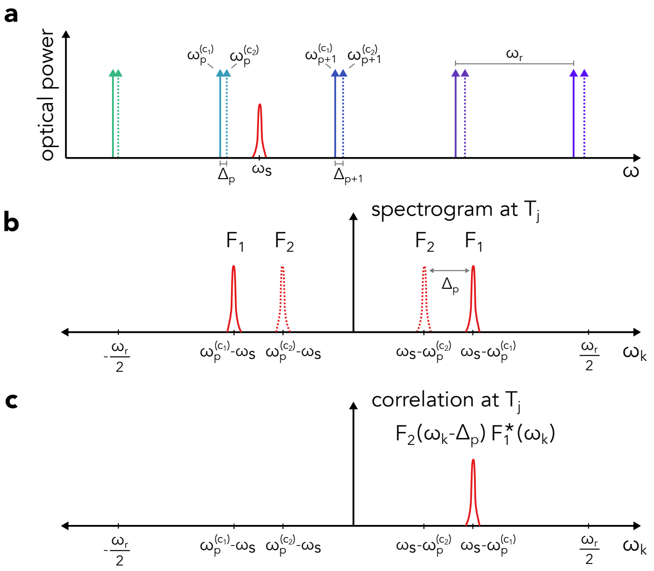

Supplementary Figure 1. Ptychoscopic reconstruction of a single-line signal. At a given slow time the spectrograms consist of a pair of delta functions (one at positive frequencies and one at negative frequencies). Their mutual phase rotates. By shifting one by the dual-comb spacing and computing the product, the negative frequency component vanishes, leaving only a result proportional to the local spectrum.

with a single comb pair $\omega_p^{(c_1)}$ and $\omega_p^{(c_2)}$ (Supplementary Figure 1a). Assuming once again that the signal is an integer multiple of the RBW away from the comb 1 line, equation (4) becomes

$$\begin{aligned} F_1(\omega_k, T_j) &= \frac{1}{N_t}\sum_i E_p^{(c_1)*} E_s e^{i(\omega_s - \omega_p^{(c_1)} - \omega_k)t_i} \\ &= E_p^{(c_1)*} E_s\, \delta(\omega_k + \omega_p^{(c_1)} - \omega_s) \quad (15) \\ F_2(\omega_k, T_j) &= \frac{1}{N_t}\sum_i E_p^{(c_2)*} E_s e^{i(\omega_s - \omega_p^{(c_2)} - \omega_k)t_i} e^{-i\Delta_p T_j} \\ &= E_p^{(c_2)*} E_s\, \delta(\omega_k + \omega_p^{(c_2)} - \omega_s) e^{-i\Delta_p T_j}. \quad (16) \end{aligned}$$

In other words, each spectrogram is a delta function at the beat frequency. Because we have posed the problem discretely, here it is Kronecker. Additionally, they have a mutual phase difference that rotates at the dual-comb difference frequency. Note that for the signal to be real, we must strictly also consider a negative frequency contribution ($E_s^* e^{-i\omega_s t}$), which beats at negative frequencies (Supplementary Figure 1b). If we now compute the instantaneous correlation between the two spectograms, we find that

$$R(\omega_k, T_j) \equiv F_2(\omega_k - \Delta_n, T_j) F_1^*(\omega_k, T_j) \quad (17)$$

$$\begin{aligned} &= e^{-i\Delta T_j} E_p^{(c_2)*} E_s\, \delta(\omega_k + \omega_p^{(c_2)} - \Delta_p - \omega_s) \\ &\quad \times E_p^{(c_1)} E_s^*\ \delta(\omega_k + \omega_p^{(c_1)} - \omega_s) \\ &= E_p^{(c_2)*} E_p^{(c_1)} |E_s|^2\, \delta(\omega_k + \omega_p^{(c_1)} - \omega_s) e^{-i\Delta_p T_j}. \end{aligned} \quad (18)$$

As we shifted F_2 towards positive Δ_n, the negative frequency contributions will vanish—the two delta functions at negative frequencies are shifted apart (Supplementary Figure 1c), resolving the double sideband ambiguity. The comb line ambiguity is resolved by Fourier transforming the result in the T direction, obtaining

$$\begin{aligned} C_n(\omega_k) &\equiv \mathcal{F}_T[R(\omega_k, T_j)](-\Delta_n) \\ &= E_p^{(c_2)*} E_p^{(c_1)} \left[|E_s|^2 \, \delta\left(\omega_k - (\omega_s - \omega_p^{(c_1)})\right) \delta_{np} \right], \end{aligned} \tag{19}$$

provided that data has been recorded for an integer number of $1/\Delta f_r$'s. Thus, we have correctly determined that the signal spectrum is a delta function offset from the pth line by $\omega_s - \omega_p^{(c_1)}$ (and only the pth line), and we have correctly determined that its magnitude is $|E_s|^2$.

Note that the prior analysis says nothing about the bandwidth of the detector. For dense combs, multiple pairs of comb lines can beat with the signal. In this case, each pair of combs will actually yield a competing version of the same data, albeit at different intermediate frequencies—a Rashomon effect. In the single line case shown above, the $(p+1)$th line would yield a delta function at $\omega_s - \omega_p^{(c_1)} - \omega_r$ (a large negative frequency). However, as detector responsivity falls at high IFs, the data corresponding to beating with the nearest comb line will typically be the most reliable.

SUPPLEMENTARY NOTE 2: SENSITIVITY

For astronomical applications, it is important to analyze the sensitivity of this approach. We do so for the case of additive white Gaussian noise (AWGN) and compare with the sensitivity of a tunable local oscillator. We assume that each detector measurement $S_i(t)$ is perturbed by a white noise source with variance σ^2. This noise source is also taken to be uncorrelated between each detector. By propagating this noise through the reconstruction, one can show that the variance of the extracted signal in the dual comb version is given by

$$\begin{aligned} \mathrm{Var}\left[\mathrm{Re}\, C_n(\omega)\right] &= \frac{1}{2N_T}\left(\frac{\sigma^2}{N_t}\right)^2 \\ &+ \frac{\sigma^2}{2N_T N_t} \sum_m \left|E_m^{(c_1)}\right|^2 P_s(\omega_m^{(c_1)} + \omega) \\ &\qquad + \left|E_m^{(c_2)}\right|^2 P_s(\omega_m^{(c_2)} - \Delta_n + \omega) \end{aligned} \tag{20}$$

As an example, in Supplementary Figure 2 we plot this expression for the data plotted in Figure 3. Because the summation over m is over both positive and negative frequencies, this means that the noise at a single frequency comes from the double-sided power spectral density of all signal components that share an IF with the frequency under consideration. The noise is frequency-dependent

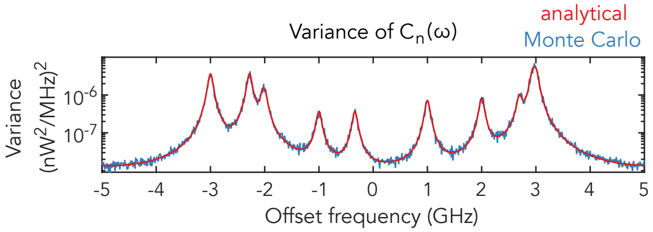

Supplementary Figure 2. Variance of the reconstruction in Figure 3 for white noise of RMS power 10 nW. This was evaluated analytically using Supplementary Equation (20) and numerically using Monte Carlo.

and resembles the sum of the two raw power spectral densities. This is practically speaking a dynamic range limitation: if one attempts to measure a weak signal that shares an IF with a much stronger signal, image noise generated by the stronger signal will swamp the weaker one. Whether this is tolerable depends on the source and on the application. For spectra consisting of relatively narrow lines it can be avoided by a proper choice of f_r, for example.

The corresponding expression for using a single LO with electric field $E_0 e^{i\omega_0 t} + c.c.$ to measure double sideband intensity is

$$\begin{aligned} \mathrm{Var}\left[I_0(\omega_0)\right] &= \frac{1}{N_T}\left(\frac{\sigma^2}{N_t}\right)^2 \\ &+ \frac{2\sigma^2}{N_T N_t} \left|E_0\right|^2 \left(P_s(\omega_0 + \omega) + P_s(\omega_0 - \omega)\right) \end{aligned} \tag{21}$$

Both expressions have a constant term independent of the signal—usually neglected since it is fourth-order in the noise—and both have a term that decreases linearly with the total measurement time. To compare these two expressions, we note that for a uniform comb the measurement time of the tunable LO must be N_c times smaller to account for the fact that the comb measurement is multiplexed. In addition, the power per comb tooth for the comb version should be N_c times smaller than the tunable LO's power if the mixer is to be optimally pumped. When there are M overlapping lines in the IF, the signal-to-noise ratio (SNR) for both the comb and the tunable LO are respectively given by

$$\mathrm{SNR}^2_{\mathrm{comb}} = \frac{1}{M} \frac{N_T N_t}{N_c} \frac{1}{2\sigma^2} \left|E_0\right|^2 P_s(\omega_n^{(c_1)} + \omega) \tag{22}$$

$$\mathrm{SNR}^2_{\mathrm{tuned}} = \frac{N_T N_t}{N_c} \frac{1}{2\sigma^2} \left|E_0\right|^2 P_s(\omega_0 + \omega) \tag{23}$$

In the best case scenario, where no lines overlap, the SNRs are identical. In the worst case scenario (a broadband light source), $M = N_c$ and the SNR is a factor of $\sqrt{N_c}$ times worse.

In addition to additive noise, there is multiplicative noise that arises from the fact that even incoherent signals can have transient frequency domain correlations.

Even when $\langle E_s(\omega)E_s^*(\omega+n\omega_r)\rangle = 0$, it will not be the case that $\left\langle |E_s(\omega)E_s^*(\omega+n\omega_r)|^2\right\rangle = 0$. This manifests as noise, and it effectively limits the dynamic range of the measurement. While the exact form depends on the details of the source—white frequency noise differs from flicker noise, for example—the variance is typically proportional to Supplementary Equation (20) for sufficiently broadband spectra.

SUPPLEMENTARY NOTE 3: PHASE CORRECTION

In the derivation of this approach we assumed that the comb lines being used to probe the signal were free of phase noise. In fact, this assumption can be relaxed if an additional dual comb spectroscopy measurement is performed to measure their mutual phase fluctuations and compensate for them. Suppose that the dual comb beating of pair n has been digitized and is given by $V_n(t) = E_n^{(c_2)}E_n^{(c_1)*}e^{i\Delta_n t}$, and that its magnitude has been divided out to construct $p_n(t) = e^{i(\phi_{2n}-\phi_{1n})}e^{i\Delta_n t}$. The correlation function is then given by

$$\begin{aligned} C_n(\omega) &= \mathcal{F}_T[F_2(\omega-\Delta_n,T)F_1^*(\omega,T)](-\Delta_n) \\ &= \frac{1}{N_T N_t}\sum_{i,j} e^{i\Delta_n(T_j+t_i)}e^{-i\omega t_i}S_2(t_i,T_j)F_1^*(\omega,T_j). \end{aligned} \tag{24}$$

Since the total time is given by $t = t_i + T_j$, the explicit dependence on Δ_n can be removed by substituting in $p_n e^{i(\phi_{1n}-\phi_{2n})}$ and noting that the summation over i is merely an FFT over the t_i axis:

$$\begin{aligned} C_n(\omega) &= \frac{1}{N_T N_t}\sum_{i,j} p_n e^{i(\phi_{1n}-\phi_{2n})}e^{-i\omega t_i}S_2(t_i,T_j)F_1^*(\omega,T_j) \\ &= e^{i(\phi_{1n}-\phi_{2n})}\left\langle \mathcal{F}_t[p_n S_2](\omega,T)F_1^*(\omega,T)\right\rangle_T \end{aligned} \tag{25}$$

This expression is convenient since it eliminates any explicit references to Δ_n by premultiplying the signal before the spectrogram calculation. Not only does it remove phase noise, but it also makes calculation of the power spectrum more convenient, as it removes the global phase of the dual comb lines. By defining the alternative correlation function $\hat{C}_n(\omega)$ as

$$\hat{C}_n(\omega) \equiv \left\langle \mathcal{F}_t[p_n S_2](\omega,T)F_1^*(\omega,T)\right\rangle_T \tag{26}$$

we find that

$$\left\langle \hat{C}_n(\omega)\right\rangle = \left|E_n^{(c_1)}E_n^{(c_2)}\right| P_s(\omega_n^{(c_1)}+\omega). \tag{27}$$

This result guarantees that phase noise does not contribute to the reconstruction, essentially by eliminating the phase entirely. Supplementary Figure 3 illustrates this for the data in Figure 3 for free-running combs. The combs have random walk phase noise producing offset fluctuations with a 1 MHz FWHM and repetition rate fluctuations with a 1 kHz FWHM, similar to what is found in free-running QCLs. As a result, the reconstruction produced by the previous approach fails. However, using the alternative correlation defined in (26), the correct result is recovered.

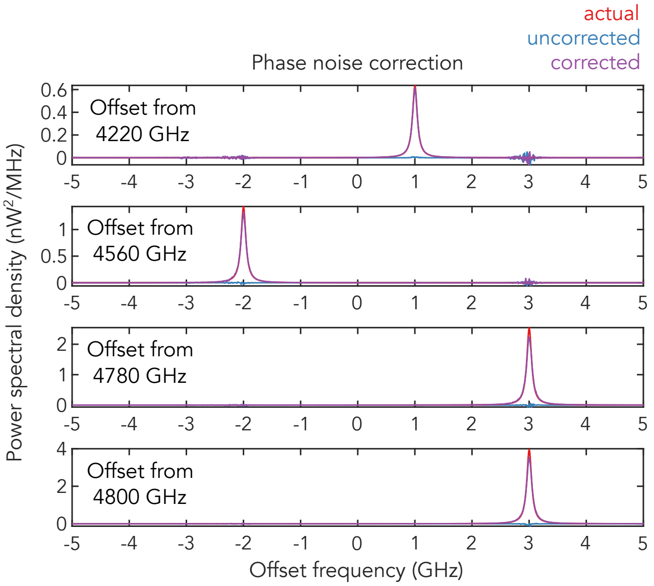

Supplementary Figure 3. Spectral reconstruction in the presence of phase noise. Offset fluctuations of 1 MHz and repetition rate fluctuations of 1 kHz preclude reconstruction, but using the alternative correlation function correctly recovers the original spectra.

SUPPLEMENTARY NOTE 4: DETECTOR NONLINEARITY

Because we are using many frequencies to reconstruct our signal, it is possible that detector/mixer nonlinearity could negatively impact the results of the measurement, for example by distorting the signal. For example, this can be a challenge in dual comb spectroscopy when highly nonlinear mixers such as hot electron bolometers are used to measure the spectrum [1], as nonlinearity will cause different comb lines to mix. However, in this case we do not expect such effects to be significant. When operating in the heterodyne limit, the measured signal is given by

$$\begin{aligned} S_i(t) &= \frac{1}{2}\left|E_{c_i}(t)+E_s(t)\right|^2 \\ &= \frac{1}{2}\left|E_{c_i}\right|^2 + E_{c_i}^* E_s + \frac{1}{2}\left|E_s\right|^2 \end{aligned} \tag{28}$$

and the $|E_{c_i}|^2$ term is much larger than the heterodyne term. Even so, it is easy to ignore because it only beats at multiples of the repetition rate (i.e., is periodic). Any nonlinearity will act upon it only to produce another signal that beats at the repetition rate, and we can therefore expect to continue to be able to ignore it.

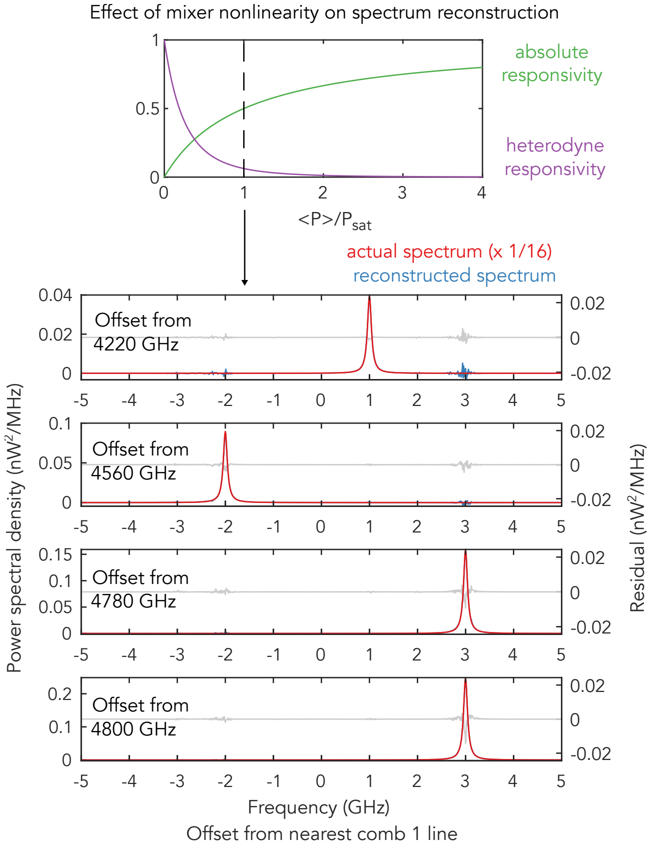

Supplementary Figure 4. Spectral reconstruction in the presence of mixer nonlinearity. Although the reconstruction is a factor of 16 times lower due to a reduction in the heterodyne sensitivity, it is otherwise unaffected.

We therefore expect that the lone effect of nonlinearity on the heterodyne measurement is to reduce its sensitivity. For example, if a standard two-level saturation model is used to model the detector response, then the output signal will be related to the input power by

$$S(t) = \frac{P(t)}{1 + \frac{P(t)}{P_{sat}}} \tag{29}$$

where P_{sat} is the saturation power. The heterodyne responsivity is essentially the differential response of this model, which is squared since both detectors suffer this decrease:

$$\left(\frac{dS}{dP}\right)^2 = \left(1 + \frac{P}{P_{sat}}\right)^{-4} \tag{30}$$

Once again, we have verified this numerically. Supplementary Figure 4 shows the result of passing the narrowband data from before through the two-level saturation model, choosing the saturation power to coincide with the average power $P_{sat} = \langle P(t) \rangle$. As expected, there is essentially no effect on the reconstruction, except that all measured reconstructed power spectral densities have been reduced by a factor of $2^4 = 16$. As with conventional heterodyne measurements, there will always be an optimal pumping power where the mixer is not yet saturated, but where the conversion gain is maximal.

SUPPLEMENTARY NOTE 5: EXPERIMENTAL SETUP

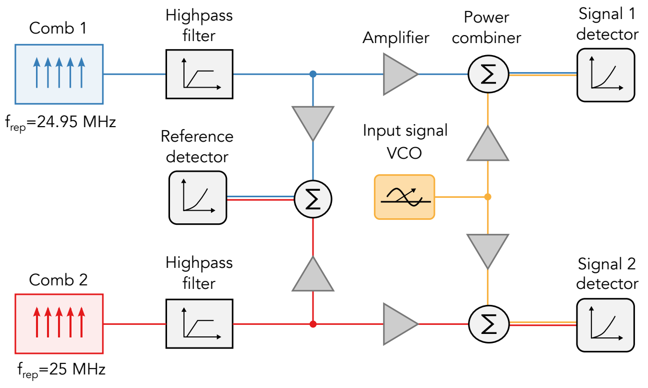

Supplementary Figure 5. Microwave experimental schematic. Microwave schematic outlining the experiment used to produce Figure 6. Unique components are labeled.

A schematic of the apparatus used to demonstrate broadband ptychoscopy is shown in Supplementary Figure 5. Two microwave frequency combs with bandwidths exceeding 4 GHz (High-Sierra Microwave) are high-pass filtered at 700 MHz to remove any low-frequency comb lines from the generated spectrum. Comb 1 has a tunable repetition rate that is set according to the desired difference frequency, while comb 2 has a fixed frequency of 5 or 25 MHz (depending on the measurement). The signal is then directed into two branches: the dual comb reference branch and the detection branch. The two branches utilize individual amplifiers since amplifiers are high directionality devices and reduce the amount of cross-talk between the detectors. In the reference branch, the two combs are summed together before being detected by a square-law detector (LTC5535ES6). This provides the reference spectrum that is used to correct the phase and amplitudes of the individual comb teeth. In the signal branch, each comb is amplified and summed with the signal to be measured and detected by a square-law detector. For simulating broadband continuous spectra, a frequency-modulated (FM) voltage-controlled oscillator (VCO) (CRYSTEK Microwave CRBV55BE) was used. To generate the FM voltage waveform a digital to analog converter (DAC) was used (Arduino Due). The DAC output was randomly generated from a designed distribution in order to ensure incoherence. Additionally, the tunable comb was set to 24.95 MHz and the 25 MHz fixed comb was used. By having a higher difference frequency a smoother spectrum is obtained. For our discrete spectrum the combs were chosen to be 4.997 MHz and 5 MHz, respectively. This was done in order to be able to achieve over 200 comb lines within a frequency octave.

Each microwave spectra displayed in the main text contains an “actual” and a “reconstructed” curve. The actual curve is generated by measuring the spectra via an RF spectrum analyzer (Agilent E4440A) at a sweep time of 1s and 100 averages. This allows us to treat this spectrum as the ground truth. The reconstructed curve was generated by measuring the voltage signal from the square-law detectors via a high-speed oscilloscope (Picoscope 6404D) and processing them according to the method derived in Supplementary Note 1. If our reconstruction method and the spectrum analyzer are set to have to have the same resolution, number of averages, and measurement time, ptychoscopy outperforms the spectrum analyzer in terms of SNR, as the non-simultaneous nature of a spectrum analyzer creates measurement artifacts that require many averages to eliminate.

SUPPLEMENTARY REFERENCES

[1] Y. Yang, D. Burghoff, D. J. Hayton, J.-R. Gao, J. L. Reno, and Q. Hu, Terahertz multiheterodyne spectroscopy using laser frequency combs, Optica **3**, 499 (2016).
